# Familial early-onset hyperuricemia and gout associated with a newly identified dysfunctional variant in urate transporter *ABCG2*

**DOI:** 10.1186/s13075-019-2007-7

**Published:** 2019-10-28

**Authors:** Yu Toyoda, Kateřina Pavelcová, Martin Klein, Hiroshi Suzuki, Tappei Takada, Blanka Stiburkova

**Affiliations:** 1 Department of Pharmacy, The University of Tokyo Hospital, Tokyo, Japan; 2 Institute of Rheumatology, Na Slupi 4, 128 50 Prague 2, Czech Republic; 3 Department of Rheumatology, First Faculty of Medicine, Charles University, Prague, Czech Republic; 4 Department of Pediatrics and Adolescent Medicine, First Faculty of Medicine, Charles University and General University Hospital, Prague, Czech Republic

## Key message

Genetic dysfunction of ABCG2 is an important risk factor of familial early-onset hyperuricemia and gout.

## Main text

We herein report the case of one European family with early onset of hyperuricemia/gout of which female proband was found to have pediatric-onset hyperuricemia associated with a newly identified functionally null variant allele in *ATP-binding cassette transporter G2* (*ABCG2*). Hitherto, we and other groups revealed that the dysfunction of ABCG2—a physiologically important urate exporter expressed in the kidney and intestine—raises the risk of hyperuricemia/gout [1–4]; however, there is little information on this relationship in terms of familial history of early-onset hyperuricemia/gout. Our case will emphasize the importance of *ABCG2* genotyping in the risk estimation of early onset of such excess urate-related diseases, which has the potential for clinical application in precision medicine.

**Fig. 1:**
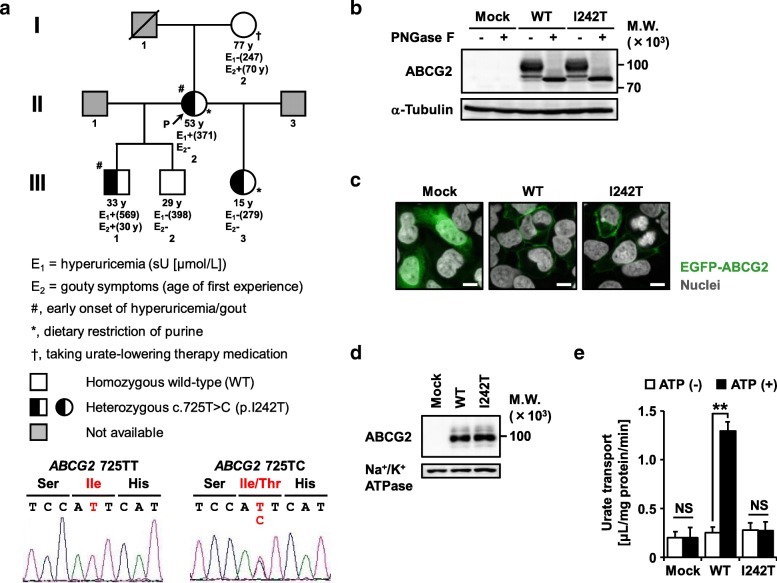
Identification and functional validation of a novel dysfunctional variant p.I242T (c.725T>C) in urate transporter ABCG2 in a family with early-onset hyperuricemia and gout. **a** Pedigree of a Czech family with early-onset hyperuricemia and gout. Electropherograms of partial sequences of *ABCG2* show the heterozygous point mutation (c.725T>C) found in the present study; each image was a representative result. P, proband; y, years old; sU, serum urate. Hyperuricemia was defined as sU levels more than 420 μmol/L (for men) or 360 μmol/L (for women and children under 15 years) on two repeated measurements, taken at least 4 weeks apart. **b** Immunoblot of whole cell lysate samples. α-Tubulin, a loading control. WT, wild-type. **c** Intracellular localization. Confocal microscopic images were obtained 48 h after the transfection. Nuclei were stained with TO-PRO-3 iodide (gray). Bars, 10 μm. **d** Immunoblot of plasma membrane vesicles. Na^+^/K^+^ ATPase, a loading control. All analytical samples were prepared from transiently ABCG2-expressing 293A cells 48 h after plasmid transfection (**b**–**d**). **e** Urate transport activities. Data are expressed as the mean ± SD. *n* = 4. Statistical analyses for significant differences were performed using a two-sided *t*-test (**, *P* < 0.01; NS, not significantly different between groups)

The pedigree is depicted in Fig. 1a. Detailed information on each subject and related methods are available in Additional file 1. Metabolic investigation for purine metabolism suggested that hyperuricemia in two patients (the family proband II:2 and III:1) was not caused by excess production of uric acid, which led us to focus on the excretion system for urate from the body.

To explore the possible causes of this familial hyperuricemia/gout, we addressed *ABCG2* genotypes in this family since dysfunction of ABCG2 is the strongest genetic risk factor of hyperuricemia/gout that affects urate excretion. As a result of targeted exon sequencing of *ABCG2*, two non-synonymous allelic variants of *ABCG2*—c.34G>A (p.V12 M) and c.725T>C (p.I242T, a novel variant)—were found in this family. There were no already-known genetic risk factors for hyperuricemia/gout such as *ABCG2* c.376C>T (p.Q126X) and c.421C>A (p.Q141K). Given that p.V12M variant that was only found in the subject III:2 reportedly has no effects on the expression and urate transport activity of ABCG2 [1], we focused on the c.725T>C (p.I242T) in each subject (Fig. 1a). Heterozygous mutation at c.725 T>C was identified in all early-onset hyperuricemia/gout patients (II:2 and III:1) and one young girl (III:3). She has been on very strict purine/lactose/gluten diet for more than 10 years, which might suppress the elevation of her serum urate (sU) levels. Moreover, subjects I:2 (post-menopausal hyperuricemia woman) and III:2 (generally healthy man) who never showed clinical signs of early onset of hyperuricemia/gout were homozygous of *ABCG2* wild-type. Thus, it is conceivable that *ABCG2* c.725T>C (p.I242T) associated with the development of early onset of hyperuricemia/gout in this family.

Next, we experimentally investigated the effect of this novel non-synonymous mutation (p.I242T) on the intracellular processing and function of ABCG2 protein. A series of biochemical analyses using transiently ABCG2-expressing mammalian cells demonstrated that the p.I242T variant had little effect on the protein level and *N*-glycosylation status of ABCG2 (Fig. 1b) and that, like ABCG2 wild-type, matured-p.I242T variant localized on the plasma membrane as a glycoprotein (Fig. 1c, d). However, functional assay revealed that contrary to the wild-type, the p.I242T variant had no ATP-dependent urate transport activity (Fig. 1e). Moreover, a cell-based urate transport assay supported that the p.I242T variant could hardly excrete urate from cells to extracellular spaces (Additional file 1: Figure S1). Thus, we concluded that the ABCG2 p.I242T variant is functionally null as an ATP-dependent urate transporter. Considering that a conserved H243 (a neighbor of I242) coordinates the γ-phosphate of ATP together with Q211 and Q126 [5], structural modification caused by the local amino acid substitution (p.I242T) might affect ATP-driven conformational changes in ABCG2, resulting in the disruption of its transport activity.

In summary, we identified a novel functionally null variant of ABCG2 that related to the development of early onset of hyperuricemia/gout in a European pedigree. To the best of our knowledge, this is the first report of pedigree analysis through three generations supporting a positive relationship between familial hyperuricemia/gout history and dysfunctional allele of *ABCG2*.

## Supplementary information

**Additional file 1.** Supplementary data

## Data Availability

The datasets used and/or analyzed during the current study are available from the corresponding author on reasonable request.

## Abbreviations

ABCG2: ATP-binding cassette transporter G2
sU: Serum urate
WT: Wild-type

## References

[1] Matsuo H, Takada T, Ichida K, Nakamura T, Nakayama A, Ikebuchi Y (2009). Common defects of ABCG2, a high-capacity urate exporter, cause gout: a function-based genetic analysis in a Japanese population. Sci Transl Med.

[2] Woodward OM, Kottgen A, Coresh J, Boerwinkle E, Guggino WB, Kottgen M (2009). Identification of a urate transporter, ABCG2, with a common functional polymorphism causing gout. Proc Natl Acad Sci U S A.

[3] Toyoda Y, Mancikova A, Krylov V, Morimoto K, Pavelcova K, Bohata J (2019). Functional characterization of clinically-relevant rare variants in ABCG2 identified in a gout and hyperuricemia cohort. Cells..

[4] Stiburkova B, Pavelcova K, Pavlikova M, Jesina P, Pavelka K (2019). The impact of dysfunctional variants of ABCG2 on hyperuricemia and gout in pediatric-onset patients. Arthritis Res Ther.

[5] Manolaridis I, Jackson SM, Taylor NMI, Kowal J, Stahlberg H, Locher KP (2018). Cryo-EM structures of a human ABCG2 mutant trapped in ATP-bound and substrate-bound states. Nature..

