# Supplementary material for: Familial early-onset hyperuricemia and gout associated with a newly identified dysfunctional variant in urate transporter ABCG2
Source: Arthritis Res Ther. 2019 Oct 28;21:219. doi: 10.1186/s13075-019-2007-7 (PMC6819377; doi:10.1186/s13075-019-2007-7)
Supplement: Supplementary file 1 — Additional file 1. Supplementary data [file 13075_2019_2007_MOESM1_ESM.docx]

**Additional File: Supplementary Data**

**Familial early-onset hyperuricemia and gout associated with a newly identified dysfunctional variant in urate transporter *ABCG2***

Yu Toyoda, Kateřina Pavelcová, Martin Klein, Hiroshi Suzuki, Tappei Takada, and Blanka Stiburkova

Correspondence to Blanka Stiburkova (stiburkova@revma.cz)

**Supplementary Information**

**Supplementary Methods**

**Supplementary References**

**Supplementary Figure S1 and S2**

**Supplementary Information**

**Subjects**

Two affected subjects and three family members belonging to a Czech family diagnosed with familial early-onset hyperuricemia/gout were studied. Written informed consent was obtained from each subject upon enrollment in this study. All tests were performed in accordance with standards set by the institutional ethics committees, which approved the project in Prague (no.6181/2015).

Hyperuricemia was defined as serum urate (sU) levels more than 420 μmol/L (for men) or 360 μmol/L (for women and children under 15 years) on two repeated measurements, taken at least four weeks apart. The definition of hyperuricemia was described in the main text. Gouty arthritis was diagnosed according to the American College of Rheumatology criteria: [1] the presence of sodium urate crystals seen in the synovial fluid using a polarized microscope or [2] at least six of 12 clinical criteria being met (1).

The family proband (II:2) is a 53-year-old woman who was found to have hyperuricemia and “bumps” in the hands and temporal areas at age of nine years; due to this pediatric-onset hyperuricemia, she was recommended a strict low-purine diet. Since then, she has never experienced remarkable hyperuricemia and acute gout attack. Her son (III:1), a 33-year-old man who experienced the first gout attack at the age of 30 years in the first metatarsal joint, suffers from recurrent gout attacks. The details will be described below.

Pediatric-onset of hyperuricemia/gout is sometimes associated with inherited metabolic diseases characterized by purine overproduction which results in the increase of uric acid synthesis (2). Hence, to examine whether hyperuricemia in our subjects could be related to the abnormality of purine metabolism, we conducted a metabolic investigation for purine metabolism as described previously (3). In all hyperuricemia patients, the urinary levels of hypoxanthine and xanthine (measure as a ratio to creatinine) were within reference ranges (hypoxanthine, ≤25 μmol/mmol creatinine; hypoxanthine, ≤30 μmol/mmol creatinine) as described below. Thus, we could exclude the possibility that the hyperuricemia was resulted from purine metabolic disorders associated with pathological concentrations of sU such as the reduced activity of hypoxanthine-guanine phosphoribosyltransferase and super-activity of phosphoribosyl pyrophosphate synthetase 1. In other words, these results suggested that hyperuricemia in two patients (II.2 and III.1) was not caused by excess production of uric acid.

***Subject II:2 (proband)***

A 53-year-old woman who was seen on rheumatology clinic for intermittent pain of distal interphalangeal joint of the hands. The pain had non-inflammatory characters, with clear clinical sings of hand osteoarthritis and corresponding changes on X-ray. However, when the medical history was explored, she referred that at the age of nine years, she was found to have hyperuricemia and “bumps” in hands and temporal area; she was recommended a strict low-purine diet. Unfortunately, neither any information on her sU levels nor medical reports from that period are currently available. Nonetheless, since then, she never experienced remarkable hyperuricemia and acute gout attack. Moreover, she told us that her mother (I:2) is treated for hyperuricemia and her son (III:1) suffers from recurrent gout attacks; this is the reason why we addressed on this family. In laboratory findings, slightly-elevated level of urate (371 μmol/L) and low level of vitamin D were detected; the excretion fraction of uric acid (EF-UA) was 7.9%. Urinary excretion of xanthine was 2.3 μmol/mmol creatinine; hypoxanthine was 0.7 μmol/mmol creatinine. An X-ray imaging showed incipient coxarthrosis and discrete changes of osteoarthritis of the hands only. Musculoskeletal ultrasound of the soles showed subcutaneous deposits of mixed echogenicity without Doppler signal (**Supplementary Figure S2**).

***Subject I:2 (the mother of the proband)***

A 77-year-old woman with medical history of gonarthrosis, dyslipidemia, and low level of vitamin D. Approximately 10 years ago, she was found to have hyperuricemia (sU values are not available) at routine medical control, and then she has been treated with allopurinol (100 mg/day). At clinical examination, she presented pain in her both knees, which was typical symptom of osteoarthritis. There were no clinical signs of gout and she never experienced acute gout attack. In laboratory tests, normouricemia (247 μmol/L), hyperlipidemia, and mild signs of chronic renal insufficiency were found; EF-UA was 4.7%. Urinary excretion of xanthine was 23.6 μmol/mmol creatinine; hypoxanthine was 7.9 μmol/mmol creatinine; oxypurinol was 22.8 μmol/mmol creatinine, which were due to the allopurinol treatment.

***Subject III:1 (the first son of the proband)***

A 33-year-old man with hypertension suffered from recurrent acute gout attacks. He is a smoker (approximately 20 cigs. /day) and drinks 3-4 L of beer daily. At the age of 15 years, high level of his sU was accidentally found, but no respective measures were taken. At the age of 30 years, he experienced the first gout attack in the first metatarsal joint. Since then, gout attack repeats approximately twice in a year. He was recommended hypouricemic treatment, but he was not adhered to medication. When he visited on our hospital, no clinical or radiographic signs of gout (chronic or acute) were found; EF-UA was 3.0%. However, hyperuricemia (569 μmol/L), hyperglycemia (5.9 mmol/L), and low level of vitamin D were found in laboratory analyses. Urinary excretion of xanthine was 0.6 μmol/mmol creatinine; hypoxanthine was 1.1 μmol/mmol creatinine.

***Subject III:2 (the second son of the proband)***

A 29-year-old man. He was generally healthy and asymptomatic. In clinical examination, no pathologies were observed; EF-UA was 3.5%. In laboratory tests, normouricemia (398 μmol/L) and low level of vitamin D were found. Urinary excretion of xanthine was 0.7 μmol/mmol creatinine; hypoxanthine was 1.1 μmol/mmol creatinine.

***Subject III:3 (the daughter of the proband)***

A 15-year-old girl. At new-born age, due to congenital double kidney and ureteral atresias, she underwent the surgical repair of the bladder; later regularly check-ups found no pathology in renal functions. Having a substantial lactose and gluten intolerance which was remarked in usual care, she has been on a very strict purine/lactose/gluten diet for more than 10 years. She had no problems with musculoskeletal system and never experienced joint pain. At clinical examination, there were normal findings; laboratory tests showed the border-line elevation of bilirubin in her blood; EF-UA was 6.0%. Regarding sU, she had a normal level (279 μmol/L). Urinary excretion of xanthine was 6 μmol/mmol creatinine; hypoxanthine was 3.9 μmol/mmol creatinine.

**Supplementary Methods**

**Sequence analysis**

ABCG2 coding regions were analyzed from genomic DNA, as described previously (4). The reference sequence was defined as version ENST00000237612.7 (location: Chromosome 4: 88,090,269−88,158,912 reverse strand) (www.ensembl.org).

**Clinical and biochemical investigations**

Urate and creatinine levels were measured as described previously (3) using a specific enzymatic method and the Jaffé reaction adapted for an auto-analyzer (Hitachi Automatic Analyzer 902, Roche), respectively. High performance liquid chromatography determination of hypoxanthine, xanthine and oxypurinol in urine was carried out using a high-performance liquid chromatography-photodiode array system (Waters Alliance 2695 and Photodiode Array Detector 2998; Waters Corp., Milford, MA, USA) according to our previous study (3, 5).

**Materials**

ATP, AMP, creatine phosphate disodium salt tetrahydrate, and creatine phosphokinase type I from rabbit muscle were purchased from Sigma-Aldrich (St. Louis, MO, USA) and [8-^14^C]-uric acid (53 mCi/mmol) were purchased from American Radiolabeled Chemicals (St. Louis, MO, USA). All other chemicals used were commercially available and of analytical grade.

**Preparation of ABCG2 p.I242T expression vector**

To express human ABCG2 (NM_004827.3) fused with EGFP at its N-terminus (EGFP-ABCG2) and EGFP (control), we used an ABCG2/pEGFP-C1 plasmid that was from our previous study (6). Using a site-directed mutagenesis technique, an ABCG2 I242T/pEGFP-C1 plasmid were generated from an ABCG2 wild-type (WT)/pEGFP-C1 plasmid. Introduction of the mutation was confirmed by full sequencing using the BigDye Terminator v3.1 (Applied Biosystems Inc., Foster City, CA, USA) and an Applied Biosystems 3130 Genetic Analyzer (Applied Biosystems Inc.) as described previously (6).

**Cell culture and transfection**

Human embryonic kidney 293 cell-derived 293A cells were purchased from Life Technologies (Carlsbad, CA, USA) and cultured in Dulbecco’s Modified Eagle’s Medium (DMEM; Nacalai Tesque, Kyoto, Japan) supplemented with 10% fetal bovine serum (Biowest, Nuaillé, France), 1% penicillin/streptomycin, 2 mM L-glutamine (Nacalai Tesque), and 1 × Non-Essential Amino Acid (Life Technologies) at 37°C in an atmosphere of 5% CO_2_. Each vector plasmid for ABCG2 WT or p.I242T was transfected into 293A cells by using polyethyleneimine MAX (PEI-MAX; 1 mg/mL in milliQ water, pH 7.0; Polysciences Inc., Warrington, PA, USA) as described previously (7). The amount of plasmid DNA used for transfection was adjusted to be the same for ABCG2 WT and p.I242T.

**Preparation of whole cell lysates**

Forty-eight hours after the transfection, whole cell lysates were prepared in an ice-cold lysis buffer A containing 50 mM Tris/HCl (pH 7.4), 1 mM dithiothreitol, 1% (w/v) Triton X-100, and a protease inhibitor cocktail for general use (Nacalai Tesque) as described previously (8). Protein concentration of the whole cell lysate was quantified using a BCA Protein Assay Kit (Pierce, Rockford, IL, USA) with bovine serum albumin (BSA) as a standard according to the manufacturer’s protocol. For glycosidase treatment, the whole cell lysate samples were incubated with PNGase F (New England Biolabs Japan Inc., Tokyo, Japan) (1.25 U/μg of protein) at 37°C for 10 min as described previously (7), and then subjected to immunoblotting.

**Preparation of ABCG2-expressing plasma membrane vesicles**

Plasma membrane vesicles were prepared from ABCG2-expressing 293A cells as described previously (7). Obtained plasma membrane vesicles were rapidly frozen in liquid N_2_ and kept at −80°C until used. The protein concentration was measured using the BCA Protein Assay Kit.

**Immunoblotting**

Expression of ABCG2 protein in whole cell lysates and plasma membrane vesicles was examined by immunoblotting as described previously (7) with minor modifications. In brief, the prepared samples were mixed with a sodium dodecyl sulfate polyacrylamide gel electrophoresis sample buffer solution containing 10% 2-mercaptoethanol, separated by electrophoresis on poly-acrylamide gels, and then transferred to Polyvinylidene Difluoride membranes (Immobilon; Millipore Corporation, Billerica, MA, USA) by electroblotting at 15 V for 60 min. For blocking, the membrane was incubated in Tris-buffered saline containing 0.05% Tween 20 and 3% BSA (Nacalai Tesque) (TBST-3% BSA). After overnight incubation at room temperature, blots were probed with a rabbit anti-EGFP polyclonal antibody (A11122; Life Technologies; diluted 1,500 fold in TBST-0.1% BSA), a rabbit anti-α-tubulin antibody (ab15246; Abcam Inc., Cambridge, MA, USA; diluted 1,000 fold), or a rabbit anti-Na^+^/K^+^-ATPase α antibody (sc-28800; Santa Cruz Biotechnology Inc., Santa Cruz, CA, USA; diluted 1,000 fold) followed by incubation with a donkey anti-rabbit immunoglobulin G (IgG)-horseradish peroxidase (HRP)-conjugated antibody (NA934V; diluted 4,000 fold for EGFP-ABCG2 or 3,000 fold for α-tubulin and Na^+^/K^+^-ATPase). HRP-dependent luminescence was developed using the ECL^TM^ Prime Western Blotting Detection Reagent (GE Healthcare UK Ltd., Buckinghamshire, UK) and detected using a multi-imaging Analyzer Fusion Solo 4^TM^ system (Vilber Lourmat, Eberhardzell, Germany).

**Confocal laser scanning microscopic observation**

For confocal laser scanning microscopy, 48 h after the transfection, 293A cells were fixed with ice-cold methanol for 10 min and then subjected to the visualization of nuclei with TO-PRO-3 Iodide (Molecular Probes, Eugene, OR, USA) as described previously (7). To analyze the localization of EGFP-fused ABCG2 protein, fluorescence was detected using a FV10i Confocal Laser Scanning Microscope (Olympus, Tokyo, Japan).

**Urate transport assay**

The urate transport assay with ABCG2-expressing plasma membrane vesicles was conducted using a rapid filtration technique as described in our previous studies (7, 9). In brief, each plasma membrane vesicle (0.25 mg/mL) was incubated with 20 μM of radiolabeled urate in the reaction mixture (10 mM Tris/HCl, 250 mM sucrose, 10 mM MgCl_2_, 10 mM creatine phosphate, 1 mg/mL creatine phosphokinase, pH 7.4, and 50 mM ATP or AMP as a substitute of ATP) for 10 min at 37°C. The urate transport activity was calculated as an incorporated clearance (μL/mg protein/min) defined as the incorporated level of urate [DPM/mg protein/min]/urate level in the incubation mixture [DPM/μL].

**Cell-based urate transport assay**

A urate uptake assay using both URAT1 and ABCG2-expressing 293A cells was conducted according to our previous study (10) with minor modifications. In brief, 293A cells were seeded onto cell culture 12-well plates at a concentration of 0.92 × 10^5^ cells/cm^2^; 24 hours after the seeding, URAT1 WT (NM_144585.3)/pEGFP-C1 (0.5 μg) and ABCG2/pEGFP-C1 (0.5 μg) vectors were transiently transfected to the cells using PEI-MAX (total 1 μg of plasmid/5 μL of PEI-MAX/well). As a control vector, pEGFP-C1 was used. After the first 24 h of incubation, the medium was replaced with fresh medium. Forty-eight hours after the plasmid transfection, urate uptake assay was carried out using Cl^-^-free transport buffer (125 mM Na-gluconate, 4.8 mM K-gluconate, 1.2 mM KH_2_PO_4_, 1.2 mM MgSO_4_, 1.3 mM Ca-gluconate, 25 mM HEPES, 5.6 mM D-glucose, and pH 7.4) containing 10 μM [8-^14^C]-urate. In this assay, the cells were incubated for 5 min (an adequate period for the evaluation of net urate-uptake activity of the cells) at 37°C. The urate transport activity was calculated as the incorporated clearance (μL/mg protein/min).

**Statistical analysis**

All statistical analyses were performed by using EXCEL 2019 (Microsoft Corp., Redmond, WA, USA) with Statcel4 add-in software (OMS publishing Inc., Saitama, Japan). The numbers of biological replicates (*n*) were described in the figure legends. When analyzing multiple groups, the similarity of variance between groups was compared using Bartlett’s test. After passing the test for homogeneity of variance, a parametric Tukey–Kramer multiple-comparison test was used. In the case of a single pair of quantitative data, after comparing the variances of a set of data by *F*-test, unpaired Student’s *t* test was performed. Statistical significance was defined in terms of *P* values less than 0.05 or 0.01.

**Supplementary References**

1. Wallace SL, Robinson H, Masi AT, Decker JL, McCarty DJ, Yu TF. Preliminary criteria for the classification of the acute arthritis of primary gout. Arthritis Rheum. 1977;20(3):895-900.

2. Zikanova M, Wahezi D, Hay A, Stiburkova B, Pitts C, 3rd, Musalkova D, et al. Clinical manifestations and molecular aspects of phosphoribosylpyrophosphate synthetase superactivity in females. Rheumatology (Oxford). 2018;57(7):1180-5.

3. Mraz M, Hurba O, Bartl J, Dolezel Z, Marinaki A, Fairbanks L, et al. Modern diagnostic approach to hereditary xanthinuria. Urolithiasis. 2015;43(1):61-7.

4. Stiburkova B, Pavelcova K, Pavlikova M, Jesina P, Pavelka K. The impact of dysfunctional variants of ABCG2 on hyperuricemia and gout in pediatric-onset patients. Arthritis Res Ther. 2019;21(1):77.

5. Stiburkova B, Pavelcova K, Zavada J, Petru L, Simek P, Cepek P, et al. Functional non-synonymous variants of ABCG2 and gout risk. Rheumatology (Oxford). 2017;56(11):1982-92.

6. Toyoda Y, Takada T, Miyata H, Ishikawa T, Suzuki H. Regulation of the Axillary Osmidrosis-Associated ABCC11 Protein Stability by N-Linked Glycosylation: Effect of Glucose Condition. PLoS One. 2016;11(6):e0157172.

7. Toyoda Y, Mancikova A, Krylov V, Morimoto K, Pavelcova K, Bohata J, et al. Functional Characterization of Clinically-Relevant Rare Variants in ABCG2 Identified in a Gout and Hyperuricemia Cohort. Cells. 2019;8(4):363.

8. Toyoda Y, Sakurai A, Mitani Y, Nakashima M, Yoshiura K, Nakagawa H, et al. Earwax, osmidrosis, and breast cancer: why does one SNP (538G>A) in the human ABC transporter ABCC11 gene determine earwax type? FASEB J. 2009;23(6):2001-13.

9. Stiburkova B, Miyata H, Zavada J, Tomcik M, Pavelka K, Storkanova G, et al. Novel dysfunctional variant in ABCG2 as a cause of severe tophaceous gout: biochemical, molecular genetics and functional analysis. Rheumatology (Oxford). 2016;55(1):191-4.

10. Miyata H, Takada T, Toyoda Y, Matsuo H, Ichida K, Suzuki H. Identification of Febuxostat as a New Strong ABCG2 Inhibitor: Potential Applications and Risks in Clinical Situations. Front Pharmacol. 2016;7:518.

**Supplementary Figures**

**Supplementary Figure S1.**

**The effects of ABCG2 WT and I242T variant on the net uptake of urate in URAT1-expressing 293A cells.** Data are expressed as the mean ± SD. *n* = 3. Statistical analyses for significant differences were performed using Bartlett’s test, followed by a parametric Tukey–Kramer multiple-comparison test. Different letters indicate significant differences between groups (*P* < 0.01). There were no significant differences between the groups indicated by same letters (*P* > 0.05).


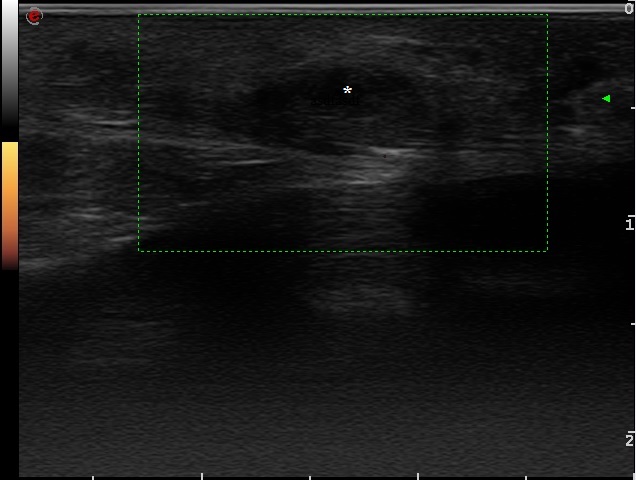


**Supplementary Figure S2.**

**Ultrasonographic scan of the plantar area of Subject II:1 (proband).** Mixed echogenic subcutaneous deposit, expressing no Doppler signal (*). The deposit is not compressible and it is not connected or communicating with the underlying tendon.
